# Drought and high nighttime temperature impact on soybean seed yield and quality under ambient and elevated CO_2_ environments

**DOI:** 10.1038/s41598-025-20392-0

**Published:** 2025-10-21

**Authors:** Naflath Thenveettil, Raju Bheemanahalli, Tulsi P. Kharel, Krishna N. Reddy, Wei Gao, K. Raja Reddy

**Affiliations:** 1 Department of Plant and Soil Sciences, Mississippi State University, 117 Dorman Hall, Box 9555, Starkville, MS 39762 USA; 2 USDA-ARS, Crop Production Systems Research Unit, 141 Experiment Station Road, P.O. Box 350, Stoneville, MS 38776 USA; 3 USDA UVB Monitoring and Research Program, Department of Ecosystem Science and Sustainability, Colorado State University, Fort Collins, CO 80523 USA

**Keywords:** High night temperature, Drought, Physiology, Seed yield, Seed oil, Fatty acids, Physiology, Plant sciences

## Abstract

The increasing prevalence of abiotic stresses, including high nighttime temperatures (HNT) and drought during the reproductive stage, poses a risk to global soybean production. Additionally, the influence of rising atmospheric CO_2_ levels must be considered when addressing changes in temperature and drought conditions. In this study, two soybean genotypes, DS25-1 and DS31-243, were grown under control (0.15 m^3^ m^–3^ volumetric soil moisture content (VWC) and 30/22 °C day/night temperature), HNT (30/26 °C day/night temperature), and drought (0.08 m^3^ m^–3^ VWC) conditions at ambient (425 ppm, aCO_2_) and elevated (725 ppm, eCO_2_) CO_2_ concentrations in sunlit plant growth chambers during flowering and pod development stages. The plants exposed to eCO_2_ under control and drought conditions showed increased photosynthesis (DS25-1: 55 and 142%, and DS31-243: 77 and 61%) and non-photochemical quenching (DS25-1: 98 and 57%, and DS31-243: 67 and 126%) compared to aCO_2_. On average, the pods and seed numbers increased by 60% and 59%, respectively, under HNT and eCO_2_ compared to HNT and aCO_2_. In contrast, the drought decreased pods and seeds by 43% across genotypes and CO_2_ environments. This has resulted in a reduction in seed yield by 62% and 56% in DS25-1 and DS31-243, respectively, under drought conditions compared to the control. Under aCO_2_, the seed yield of DS31-243 was reduced by 42% under HNT compared to the control. The seed protein content was reduced under eCO_2_, while other treatments did not influence its content. The seed carbohydrate content decreased under the HNT condition, while the drought and eCO_2_ did not influence its production. The stress conditions during seed development resulted in reduced polyunsaturation, while the oleic acid content increased. The study highlighted the positive impacts of eCO_2_ on physiology and yield.

## Introduction

The Earth’s atmosphere is constantly changing, resulting in increased severity of agricultural yield loss^1^. With the increasing global population, it is challenging to achieve food security amid the potential impact of extreme temperatures and the intensity and frequency of drought on crop productivity^2^. Increased greenhouse gas emissions, with carbon dioxide (CO_2_) emissions rising at an additional rate of 0.5% since the onset of the Industrial Revolution, have elevated the atmospheric CO_2_ concentration, [CO_2_], up to 425 ppm^3^. The addition rate of CO_2_ is currently around 2.5 ppm every year-subsequent increase in absorbed heat radiation results in rising global temperatures. On average, the global surface temperature rose by 1 °C above the pre-industrial age of 1850–1900, and 2023 was the warmest year on record^4^.

An increase in global temperatures raises the likelihood of experiencing more severe drought conditions^5^. According to the U.S. Drought Monitoring report, around 19% of the contiguous US was affected by drought during June 2024^6^. The southeast regions of the US are at particular risk since droughts occur more frequently, intensely, and are prolonged^5^. The lower Mississippi River valley experienced an increase in drought area by 3.3% at the end of June 2024^6^. Drought severely impacts agricultural production, affecting the groundwater supply and causing crop failure. It accounted for a monetary loss of 14.5 billion USD in the agricultural sector in 2012^7^. The US soybean production is at risk of economic loss since 35% of the production area is experiencing either moderate (31%) or severe (4%) drought^8^. Soybean, being a major industrial crop in the US, will have a substantial effect on the US economy due to the drought-associated damages and additional costs. Drought stress affects multiple morpho-physiological traits, such as reduced stomatal conductance, increased canopy temperature, and decreased quantum efficiency^9^, which results in reduced photosynthesis and biomass production^10^. The maximum water requirement of soybeans is typically during the reproductive stage^11,12^. Numerous studies on the effects of drought stress on the reproductive stage in soybeans have reported a yield reduction of 35%^9^ to more than 80%^13^.

Along with the yield loss, the drought during the seed developmental and filling stage adversely affects the seed quality^10,14^. Soybeans are a good source of protein, oil, and other nutrients, and they determine the market value. The deposition of oil and protein in the seeds continues until 70 days from flowering, with more significant deposition around 20–40 days after flowering^15^. Thus, changing environmental conditions during the seed-filling stage will impact its composition. Previous research has reported that the protein accumulation decreased under drought stress conditions while the oil content increased^16^. In contrast, Poudel et al.^9,17^ observed an increase in seed protein content and a decrease in seed oil content. A few other studies proposed a reduction in protein content as a result of the downregulation of genes encoding storage proteins^18^. They also reported that the drought stress increased soluble sugars and reduced the expression of genes involved in lipid biosynthesis. On the other hand, stearic, oleic, and linolenic acids increased under drought conditions, while other fatty acids decreased^16^. These reported variations could be due to the differences in the genetic architecture of genotypes used in the study and changes in the growing conditions of plants. Hence, further focus is given to studying the impact of drought stress on soybean seed quality under drought, while maintaining optimal growing conditions.

Along with the increase in average temperature over the years, the nighttime temperature in most locations is rising faster than the daytime temperature^19^, leading to a reduction in the diurnal temperature range^20^. The global daily mean minimum temperature increased by 0.8 °C per century, compared to the daily maximum temperature increase of 0.4 °C^21^. It has been reported that the HNT (29 °C) adversely affects soybean yield by reducing the number of pod sets and pollen germination^22^. A similar observation was also made by Lin et al.^23^, reporting a reduction of 4.6% yield for every  °C increase in nighttime temperature. Regarding the physiology of plants under HNT, it increased night respiration, leading to more significant carbon loss^24,25^. In addition to the reduction in photosynthesis, the HNT also causes cell membrane damage, production of reactive oxygen species, and a decrease in antioxidant capacity^26^. In rice, the HNT during the post-flowering stage increased the carbon loss compared to the pre-flowering stage^27^. A similar observation was made in soybeans by Puteh et al.^28^.

The HNT during the pod-filling period influences the seed’s chemical composition^29^. Sucrose is the primary source of energy used to develop seeds^30^. Since sucrose content decreases during HNT due to high night respiration, it severely impacts the seed quality^31^. Impa et al.^32^ reported a reduction in grain starch content and an increase in protein and lipid content in winter wheat during HNT. In soybeans, HNT resulted in smaller seed size and weight and reduced sucrose accumulation compared to protein content^31^. Further insights are required to understand the effect of HNT on the composition of other carbohydrates and fatty acids in soybeans.

The current study aims to (i) identify the drought—and HNT-driven changes in plant physiology and seed yield during flowering and seed development stages at ambient and elevated CO_2_ levels and (ii) evaluate the changes in seed chemical composition in response to drought and HNT during seed development.

## Materials and methods

The experiment was conducted in the Soil–Plant-Atmospheric-Research (SPAR) facility at the Environmental Plant Physiology Laboratory, Mississippi State University, Mississippi State, USA, during the summer of 2022. The SPAR units consist of a metal bin at the bottom to hold the root mass and a Plexiglas chamber at the top to hold the plant canopy. The units precisely maintain the designated temperature and CO_2_ levels through an automated computer control system^33^. Two soybean genotypes, DS25-1 and DS31-243, from maturity group IV, were obtained from the United States Department of Agriculture-Agricultural Research Service, Stoneville, Mississippi, and selected for the study. The study does not include human or animal subjects.

### Experimental setup

Six SPAR units were used for the experiment. Twenty polyvinyl chloride pots (15 × 46 cm) in 10 columns × 2 rows were arranged in each SPAR unit, accommodating 10 pots for each genotype (DS25-1 and DS31-243). The pots contained a hole of 0.5 cm in diameter at the bottom to drain the excess water and nutrients. Each pot was prepared with 0.25 kg of gravel at the bottom, followed by a soil mixture consisting of fine sand (particle size less than 0.3 mm) and topsoil (87% sand, 2% clay, and 11% silt) in a 3:1 ratio by volume. Four seeds from both genotypes were planted in alternate columns for randomization and were thinned to one plant per pot at the two-leaf stage. Each unit was maintained at 30/22 °C, day/night temperature up to flowering. Three SPAR units were maintained at 425 ppm CO_2_, and the other three were at 725 ppm since planting. The pots were irrigated with full-strength Hoagland’s nutrient solution^34^ three times a day. The irrigation volume was adjusted based on the daily evapotranspiration for an adequate water supply^33^ until the flowering stage.

### Experimental treatments

The soybean genotypes were subjected to drought and HNT stresses from flowering (40 days after planting) to harvest maturity (120 days after planting). Two levels of irrigation (control: 100% irrigation, 0.15 m^3^ m^–3^ volumetric soil moisture content (VWC) and drought: 50% irrigation, 0.08 m^3^ m^–3^ VWC) and high-night temperature (control: 30/22 °C and HNT: 30/26 °C) were maintained at 425 ppm (aCO_2_) and 725 ppm (eCO_2_) CO_2_ concentrations (Table 1). The SPAR unit with 100% irrigation and 30/22 *°*C, day/night temperature was a standard control for both the drought and HNT treatments. Four Decagon soil moisture sensor probes (ECH_2_O, EC-5, Decagon Devices, Inc., Pullman, WA, USA) per treatment were inserted in control and drought treatments under aCO_2_ and eCO_2_ to monitor the soil moisture content. Daytime temperatures were regulated from sunrise to sunset, with a gradual transition to nighttime temperatures occurring over 30 min after sunset. The two genotypes were subjected to six treatment combinations in the study. The experiment followed a two-factor, completely randomized design considering treatments (CO_2_, drought, and HNT combinations) and genotypes as factors with ten replications. Black mesh shade cloths were placed along the edges of the Plexiglas chambers to mimic the effect of surrounding border plants. The shade net was adjusted periodically to accommodate plant growth.

**Table 1:** CO_2_, high nighttime temperature, and drought treatment combinations used in the study.

Treatment units	CO_2_	Day/night-time temperature °C	Drought
Treatment 1	aCO_2_	30/22	100% irrigation, 0.15 m^3^ m^–3^ VWC^†^
Treatment 2	aCO_2_	30/26	100% irrigation, 0.15 m^3^ m^–3^ VWC
Treatment 3	aCO_2_	30/22	50% irrigation, 0.08 m^3^ m^–3^ VWC
Treatment 4	eCO_2_	30/22	100% irrigation, 0.15 m^3^ m^–3^ VWC
Treatment 5	eCO_2_	30/26	100% irrigation, 0.15 m^3^ m^–3^ VWC
Treatment 6	eCO_2_	30/22	50% irrigation, 0.08 m^3^ m^–3^ VWC

^†^ VWC is volumetric water content, measured by the amount of water present per unit volume of soil.

### Trait measurements

#### Plant physiology

The plant physiology measurements were recorded on an uppermost fully matured leaf (5th leaf from the top) during the seed developmental stage. The photosynthesis and fluorescence measurements were recorded using a photosynthesis system (LI-6400, LI-COR Environmental, Lincoln, NE, USA) between 11.00 and 13:00 h. All the gas exchange measurements were collected from five plants per genotype across all treatments. The conditions for leaf stabilization were 30 °C leaf temperature, 40–50% relative humidity, 425 or 725 µmol mol^–1^ CO_2_ concentration depending on the treatment, 1500 µmol m^–2^ s^–1^ photosynthetic active radiation, and 500 µmol s^–1^ flow rate. The gas exchange measurements, including photosynthesis rate (µmol CO_2_ m^–2^ s^–1^), stomatal conductance (gsw; mol H_2_O m^–2^ s^–1^), and leaf internal CO_2_ to ambient CO_2_ ratio (C_i_/C_a_), were measured simultaneously. Additionally, parameters related to the light reaction of photosynthesis, such as electron transport rate (ETR), non-photochemical quenching (NPQ), photosystem II efficiency (PhiPS2), and maximum efficiency of photosystem II at light-adapted state (F_v_′/F_m_′) were recorded.

#### Plant growth, biomass, and yield

The plants were harvested at maturity or 120 days after planting. The pods (no. plant^–1^) were recorded by counting the fully matured pods containing at least one seed. The pods were then oven-dried at 35 °C for 24 h to maintain the seed moisture content of approximately 14%. After drying, the pods were hand-threshed to collect seeds. The seeds (no. plant^–1^) were counted using the Old Mill Seed Counter (NP5056-Model 850–2, LICOR Inc., Lincoln, NE, USA). The 100 seed weight (g) and seed yield (g plant^–1^) were recorded for all the treatments. After the growth measurements, the plants were separated into leaves, stems, pods, and roots. They were then oven-dried at 80 °C for 72 h to record the dry weights (g) of the plant parts.

#### Seed quality

Five grams of seeds from each treatment in five replications were analyzed for their chemical constitution using NIRS spectroscopy (Perten DA7250 spectrometer, Perten Instruments, IL, USA;^35^). The resulting oil, protein, carbohydrate, and fatty acid contents were expressed in percentages.

### Data analysis

The data were subjected to analysis of variance using a two-factorial, completely randomized design. The analysis was performed using the ‘doebioresearch’ package in R^36^. Multiple comparison tests were carried out to identify genotype-specific responses using the least-significant difference (LSD) test. The impact of treatments in each genotype was separately analyzed using a one-way analysis of variance. The partial Eta squared (ƞ^2^) effect size with confidence interval of treatment, genotype, and their interactions were computed using the ‘effectsize’ package in R^37^ with 1000 bootstraps. The partial ƞ^2^ < 0.06 represents an effect size with a small contribution, 0.06 > partial ƞ^2^ < 0.14 represents an effect size with a moderate contribution, and a partial ƞ^2^ > 0.14 represents an effect size with a large contribution^38^. A graphical representation of the outcomes was generated using Sigmaplot 13.0 (Systat Software Inc., San Jose, CA, USA) and GraphPad Prism 8.00 (GraphPad Software, San Diego, CA, USA).

## Results

**Fig. 1:**
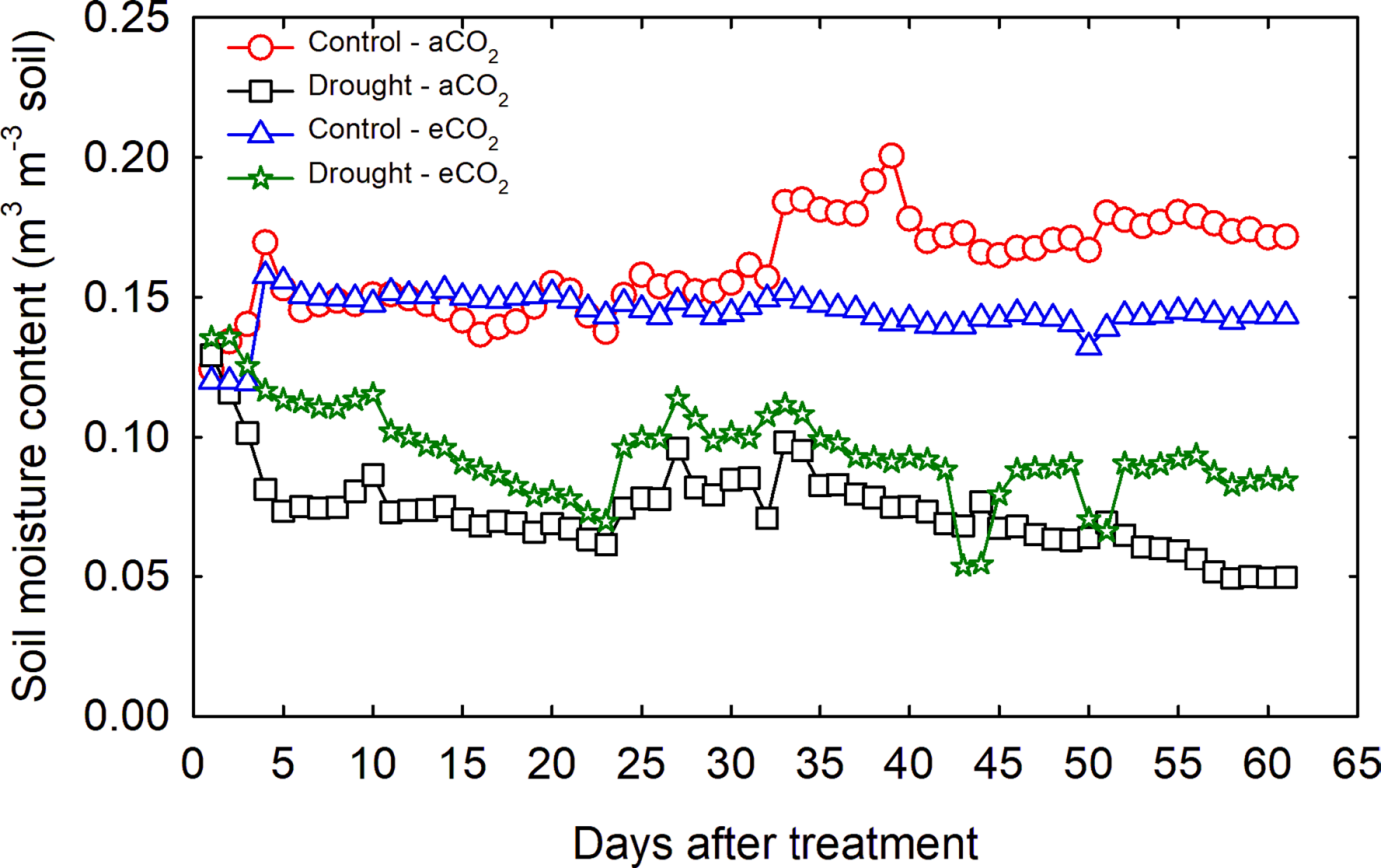
The volumetric soil moisture content of control and drought throughout the experimental period.

The average volumetric soil moisture content of the control treatment was 0.16 and 0.14 m^3^ m^–3^ soil under aCO_2_ and eCO_2_, respectively (Fig. 1). While the drought treatments maintained the soil moisture content at an average of 0.07 and 0.09 m^3^ m^–3^ soil under aCO_2_ and eCO_2_, respectively. The control and HNT treatments maintained the set day and nighttime temperatures of 30/22 °C and 30/26 °C, respectively.

### Physiology

**Fig. 2:**
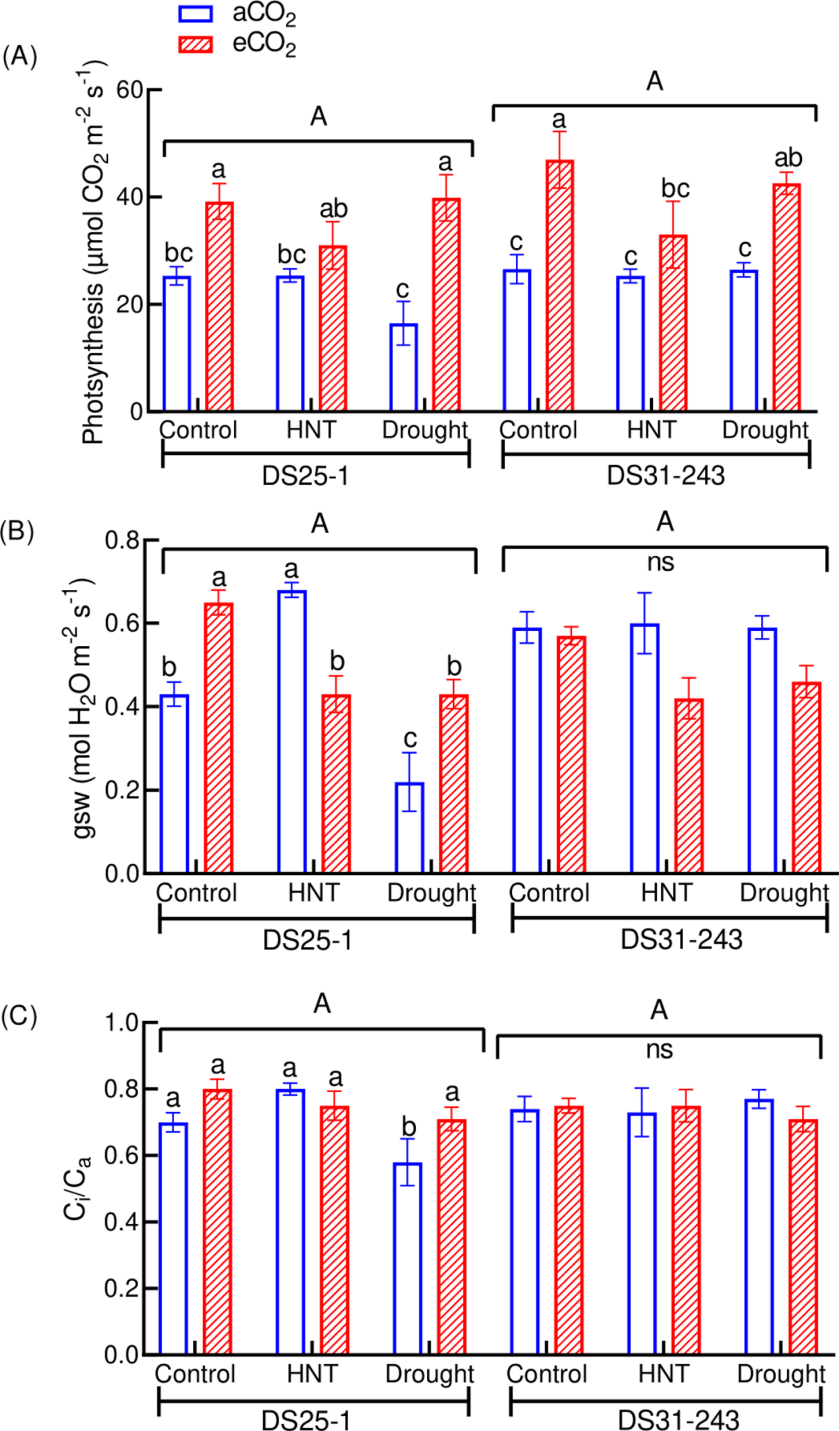
Effect of high-night temperature (HNT) and drought at ambient (aCO_2_) and elevated (eCO_2_) CO_2_ concentrations on (**A**) photosynthesis, (**B**) stomatal conductance (gsw), and (**C**) C_i_/C_a_ ratio. The upper and lowercase letters above the boxes are the least significant difference test (LSD) between genotypes and six treatments for each genotype. The treatment boxes with similar letters are statistically non-significant. ‘ns’ indicates non-significant at *p* < 0.05.

Significant notable differences were observed in photosynthesis, gsw, NPQ, and F_v_′/F_m_′ due to the treatments (Table 2). However, there were treatment × genotype interactions for all the physiological traits measured except for photosynthesis and NPQ. Based on the partial ƞ^2^ effect size, the treatments and treatment × genotype interaction showed a large effect size (> 0.14) on all the physiological traits (Table 3). While the effect of genotypes on Ci/Ca and gsw was small. These indicate that HNT and drought delivered a large contribution to the trait variation under aCO_2_ and eCO_2_ conditions. The photosynthesis of plants under HNT did not respond to the eCO_2_ environment in both genotypes. In contrast, the plants grown under control and drought conditions showed a positive response to eCO_2_ with an increase in the photosynthetic rate of 55% (control) and 142% (drought) in DS25-1 and 77% (control) and 61% (drought) in DS31-243, compared to the control and drought conditions under aCO_2_ (Fig. 2A). Under aCO_2_, gsw and C_i_/C_a_ of DS25-1 were reduced by 49% and 17% when grown under drought condition compared to control (Fig. 2B, C) while gsw under HNT conditions increased by 58% compared to control at aCO_2_. In contrast, under the same conditions, the performance of DS31-243 was comparable to that of the control and HNT.

**Table 2:** Analysis of variance (ANOVA) result of measured parameters as affected by treatments, genotypes, and their interaction.

	Treatment	Genotype	Treatment × Genotype
Physiology			
Photosynthesis	***	ns	ns
Ci/Ca	ns	ns	*
Stomatal conductance	*	ns	**
PhiPS2	ns	ns	*
ETR	ns	ns	*
NPQ	***	ns	ns
F_v_′/F_m_′	**	ns	**
Seed yield and biomass			
Shoot dry weight	***	ns	ns
Root dry weight	**	ns	**
Total dry weight	***	ns	ns
Pod number	***	***	**
Seed number	***	ns	**
Seed yield	***	ns	ns
100 seed weight	***	***	*
Seed quality			
Protein %	***	ns	***
Oil %	***	ns	***
Starch %	***	***	ns
Stachyose %	***	**	*
Sucrose %	***	***	ns
Fructose %	***	***	**
Glucose %	***	***	*
Palmitic acid %	***	ns	***
Stearic acid %	***	***	***
Linoleic acid %	***	ns	*
Linolenic acid %	*	ns	*
Oleic acid %	***	ns	***

*, **, *** represent significance level at *p* ≤ 0.05, 0.01, and 0.001, respectively, and ns is non-significant).

**Table 3:** Partial Eta-squared (ƞ^2^) effect size and confidence interval (CI) of treatments, genotypes, and their interaction on physiological traits.

Traits	Sources	Partial ƞ^2^	Lower CI	Upper CI
Photosynthesis	Treatment	0.633	0.411	0.657
	Genotype	0.095	0.000	0.252
	Treatment × Genotype	0.152	0.008	0.165
Ci/Ca	Treatment	0.244	0.004	0.269
	Genotype	0.034	0.000	0.103
	Treatment × Genotype	0.253	0.027	0.361
Stomatal conductance	Treatment	0.245	0.012	0.255
	Genotype	0.044	0.000	0.159
	Treatment × Genotype	0.190	0.020	0.195
PhiPS2	Treatment	0.241	0.014	0.254
	Genotype	0.088	0.000	0.223
	Treatment × Genotype	0.140	0.005	0.145
ETR	Treatment	0.239	0.022	0.258
	Genotype	0.090	0.000	0.249
	Treatment × Genotype	0.150	0.006	0.125
NPQ	Treatment	0.749	0.547	0.798
	Genotype	0.098	0.000	0.295
	Treatment × Genotype	0.216	0.019	0.275
F_v_′/F_m_′	Treatment	0.382	0.057	0.487
	Genotype	0.100	0.000	0.240
	Treatment × Genotype	0.227	0.026	0.292

**Fig. 3:**
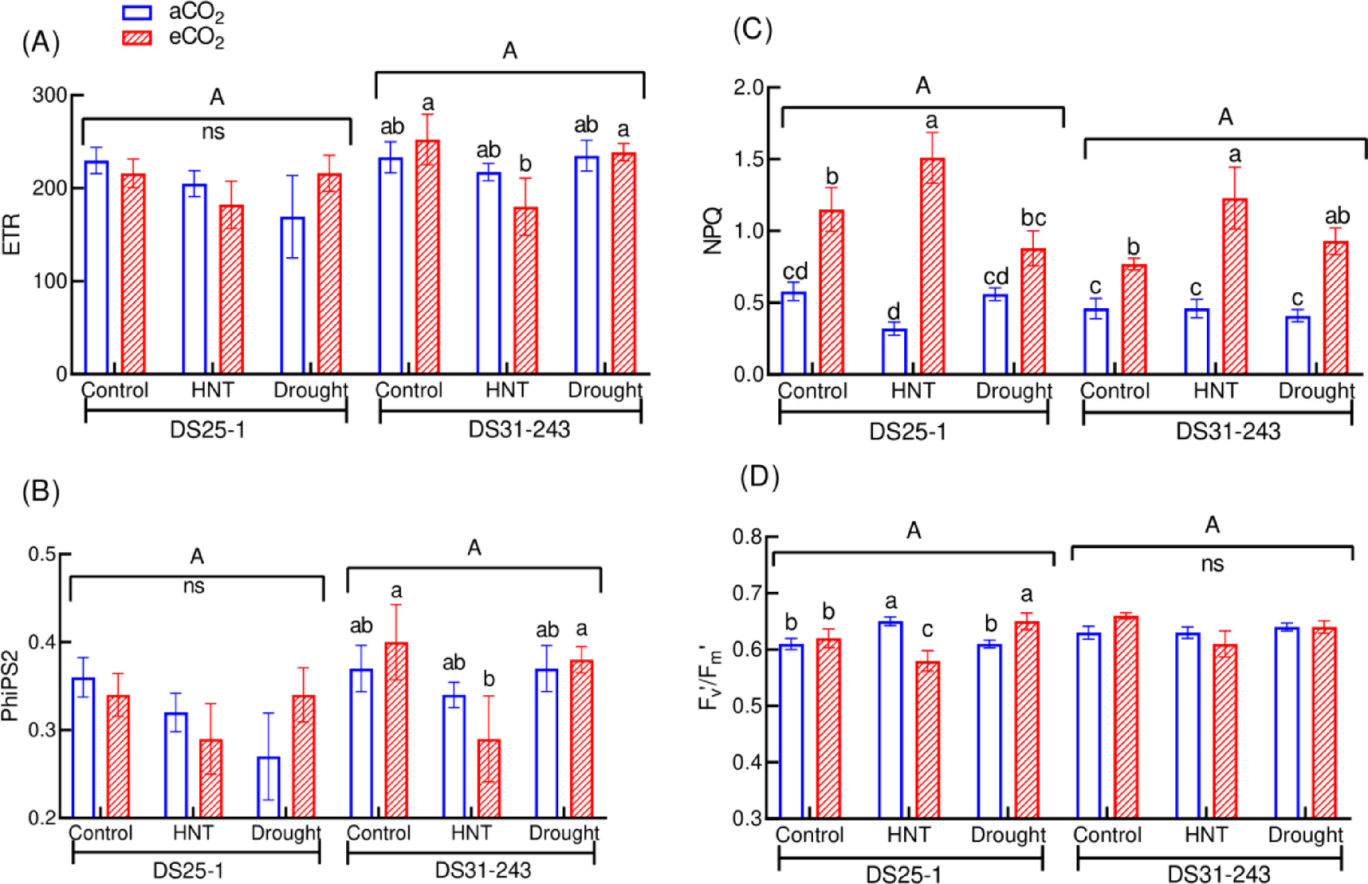
Effect of high-night temperature (HNT) and drought at ambient (aCO_2_) and elevated (eCO_2_) CO_2_ concentrations on (**A**) electron transport rate (ETR, µmol electrons m^−2^ s^−1^,  (**B**) PhiPS2, (**C**) NPQ, and (**D**) F_v_′/F_m_′. The upper and lowercase letters above the boxes are the least significant difference test (LSD) between genotypes and six treatments in each genotype. The treatment boxes with similar letters are statistically non-significant. ‘ns’ indicates non-significant at *p* < 0.05.

Among the fluorescence parameters, the ETR and PhiPS2 of plants grown under drought and HNT under aCO_2_ and eCO_2_ conditions did not change in DS25-1 (Fig. 3A, B). On the other hand, the genotype DS31-243 exhibited a reduction in ETR and PhiPS2 by 27% under HNT and eCO_2_ conditions compared to control and eCO_2_. The genotypes grown under eCO_2_ increased the NPQ by 98, 372, and 57% under control, HNT, and drought conditions in DS25-1, and 67, 167, and 126% in DS31-243, respectively, compared to the same treatment condition at aCO_2_ (Fig. 3C). In both genotypes, high NPQ was observed under HNT. A 10% reduction in F_v_′/F_m_′ was recorded in the DS25-1 genotype when grown under eCO_2_ and HNT compared to aCO_2_ and HNT, while it increased by 6% under eCO_2_ and drought compared to aCO_2_ and drought (Fig. 3D). However, the F_v_′/F_m_′ in DS31-243 was not affected by the treatments.

### Plant biomass and seed yield

**Fig.4:**
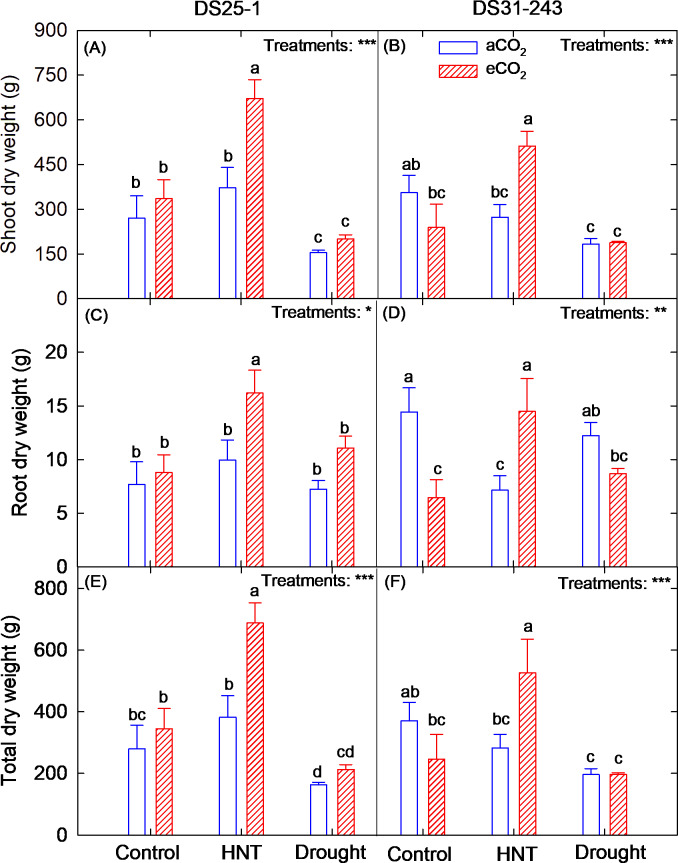
Effect of high-night temperature (HNT) and drought at ambient (aCO_2_) and elevated (eCO_2_) CO_2_ concentrations on (**A, B**) shoot dry weight, (**C, D**) root dry weight, and (**E, F**) total dry weight of DS25-1 and DS31-243 soybean genotypes. The letters above the boxes are the least significant difference test (LSD) between six treatments in each genotype. The treatment boxes with similar letters are statistically non-significant. The *, **, and *** indicate significance levels at *p* ≤ 0.05, 0.01, and 0.001, respectively, and ns indicates non-significant at *p* < 0.05.

All the plant biomass and seed yield-related parameters were influenced by the treatments (*p* < 0.01 and 0.001) (Table 2). The genotype was statistically different for pod number and 100 seed weight, while for the rest of the parameters, it was not statistically significant. The partial ƞ^2^ effect size of the treatment on biomass and yield traits was larger than the genotype and treatment × genotype interaction (Table 4). Except for pod number (0.172) and 100 seed weight (0.276), the genotype had a smaller effect size (< 0.06) on biomass and seed yield traits. In DS25-1, the above and below-ground biomass increased under HNT and eCO_2_ by 148% and 110%, respectively, compared to the control under aCO_2_, which resulted in a 145% increase in total dry weight (Fig. 4A, C, E). However, the biomass of DS31-243 under HNT and eCO_2_ was on par with that of the control under aCO_2_ (Fig. 4B, D, F). The shoot and total dry weight of DS25-1 grown under drought decreased by 43% and 42%, respectively, and of DS31-243 by 48% and 47%, respectively, compared to the control. The eCO_2_ fertilization during the reproductive stage did not influence the biomass production of both genotypes under control and drought conditions.

**Table 4:** Partial Eta-squared (ƞ^2^) effect size and confidence interval (CI) of treatments, genotypes, and their interaction on biomass and yield-related traits.

Traits	Sources	Partial ƞ^2^	Lower CI	Upper CI
Shoot dry weight	Treatment	0.532	0.240	0.626
	Genotype	0.034	0.000	0.127
	Treatment × Genotype	0.143	0.008	0.167
Root dry weight	Treatment	0.269	0.031	0.340
	Genotype	0.017	0.000	0.022
	Treatment × Genotype	0.224	0.048	0.281
Total dry weight	Treatment	0.523	0.270	0.604
	Genotype	0.034	0.000	0.123
	Treatment × Genotype	0.143	0.007	0.167
Pod number	Treatment	0.493	0.197	0.596
	Genotype	0.172	0.025	0.305
	Treatment × Genotype	0.245	0.045	0.331
Seed number	Treatment	0.514	0.249	0.620
	Genotype	0.051	0.000	0.175
	Treatment × Genotype	0.221	0.025	0.314
Seed yield	Treatment	0.384	0.140	0.479
	Genotype	0.018	0.000	0.056
	Treatment × Genotype	0.127	0.007	0.170
100 seed weight	Treatment	0.284	0.075	0.349
	Genotype	0.276	0.079	0.414
	Treatment × Genotype	0.176	0.022	0.225

**Fig. 5:**
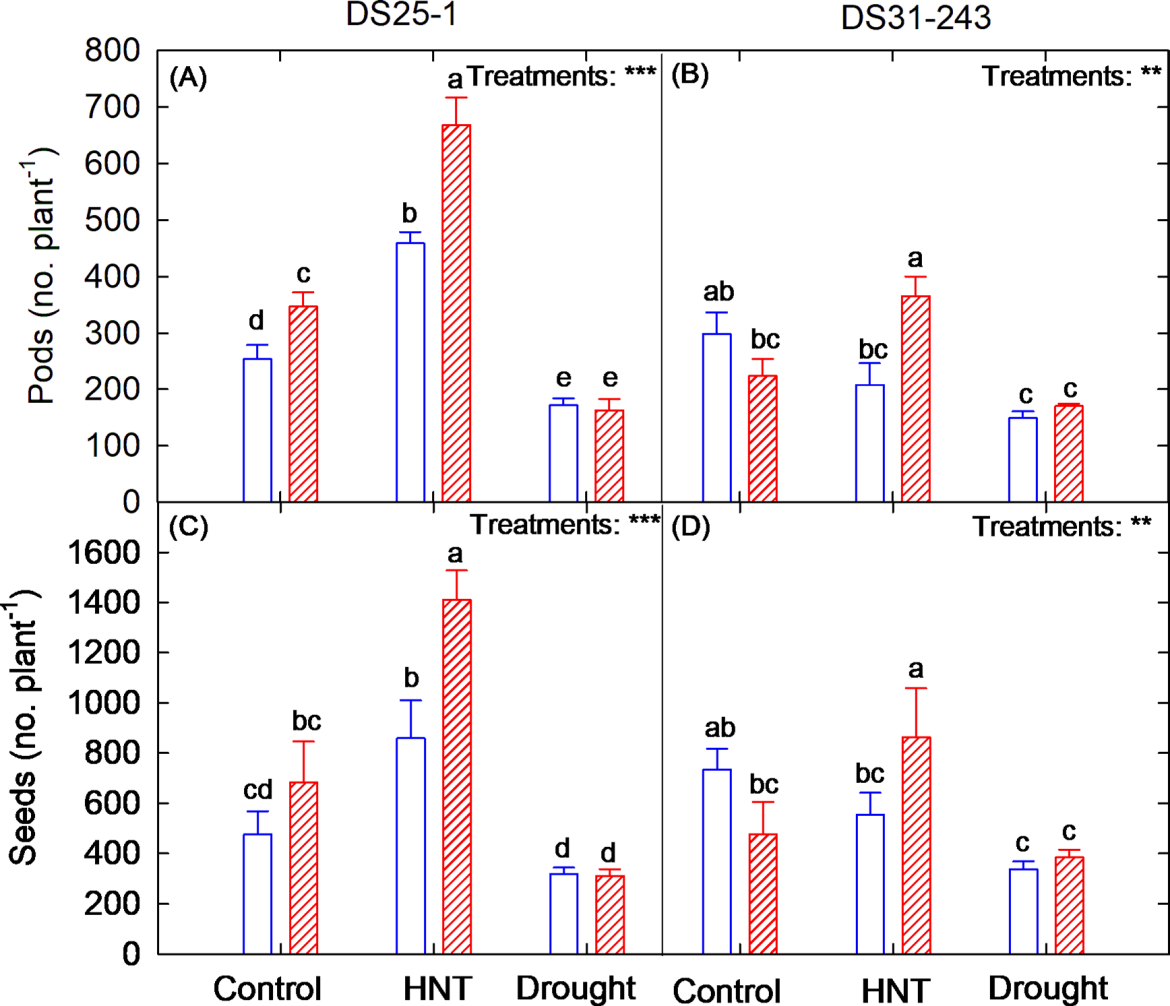
Effect of high-night temperature (HNT) and drought at ambient (aCO_2_) and elevated (eCO_2_) CO_2_ concentrations on (**A, B**) pods (no. plant^–1^) and (**C, D**) seeds (no. plant^–1^) of DS25-1 and DS31-243 soybean genotypes. The letters above the boxes are the least significant difference test (LSD) between six treatments in each genotype. The treatment boxes with similar letters are statistically non-significant. The *, **, and *** indicate significance levels at *p* ≤ 0.05, 0.01, and 0.001, respectively, and ns indicates non-significant at *p* < 0.05.

In both genotypes, maximum pods (no. plant^–1^) and seeds (no. plant^–1^) were recorded under HNT and eCO_2_ conditions (Fig. 5A–D). Under HNT, the exposure of DS25-1 to eCO_2_ increased the pods (no. plant^–1^) by 45% and seeds (no. plant^–1^) by 64%, and in DS31-243, it was 75% and 55%, respectively, compared to aCO_2_. On average, the plant’s exposure to drought conditions during the reproductive stage reduced the pods (no. plant^–1^) and seeds (no. plant^–1^) by 42% and 43%, respectively, compared to the control across the CO_2_ and genotypes.

**Fig. 6:**
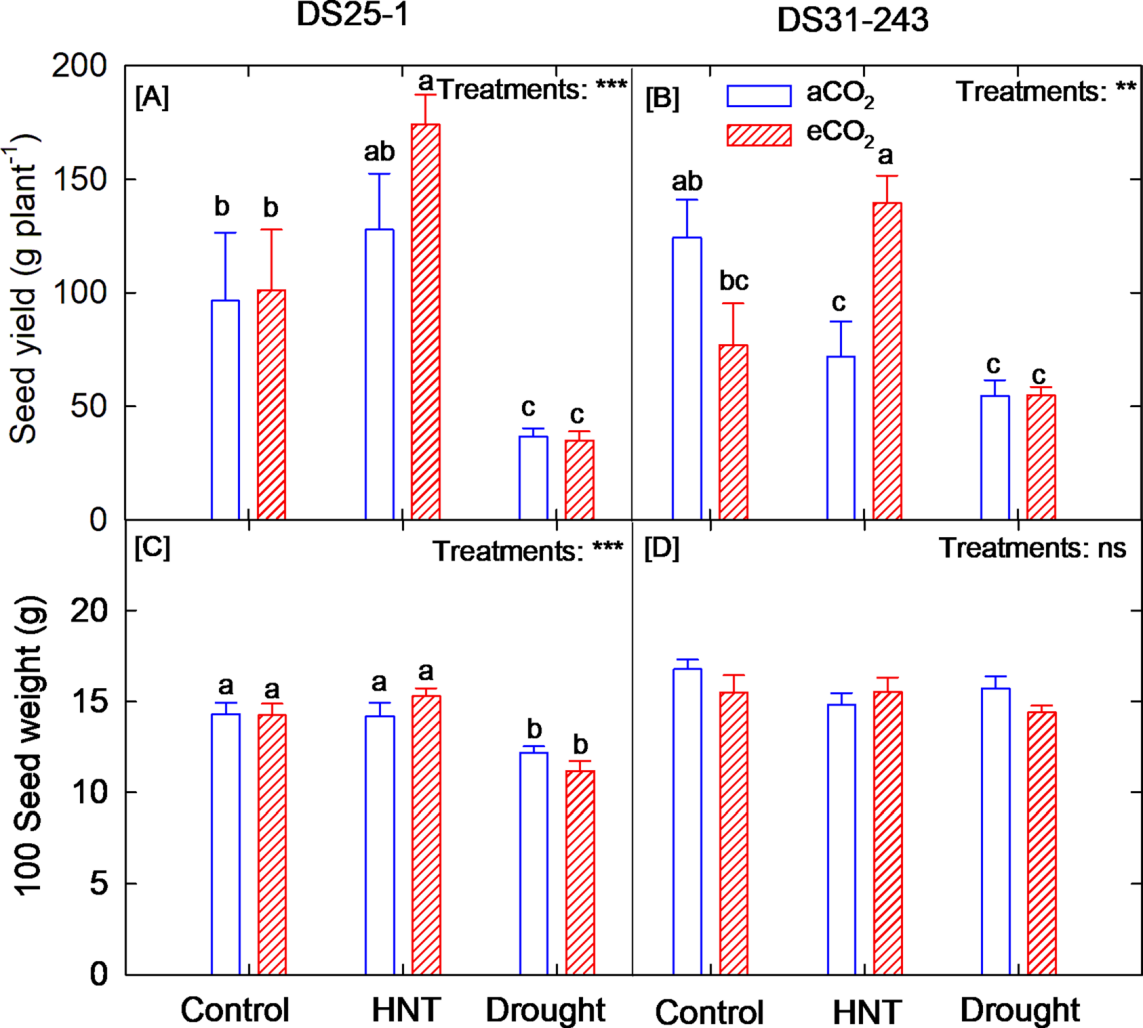
Effect of high-night temperature (HNT) and drought at ambient (aCO_2_) and elevated (eCO_2_) CO_2_ concentrations on (**A, B**) seed yield (g plant^–1^) and (**C, D**) 100 seed weight of DS25-1 and DS31-243 soybean genotypes. The letters above the boxes are the least significant difference test (LSD) between six treatments in each genotype. The treatment boxes with similar letters are statistically non-significant. The * and *** indicate significance levels at *p* ≤ 0.05 and 0.001, respectively, and ns indicates non-significant at *p* < 0.05.

With respect to seed yield, the plants grown under HNT increased the yield by 81% at eCO_2_ in DS25-1 compared to control at aCO_2_, and it was on par with the yield under HNT at aCO_2_ (Fig. 6A). On the other hand, the DS25-1 plants exposed to drought conditions reduced the yield by 62% compared to control. In the case of DS31-243, the seed yield decreased by 56% under drought conditions (Fig. 6B). While under aCO_2_, the HNT lowered the seed yield by 42% compared to the control. However, HNT under eCO_2_ had a seed yield on par with the control. A 94% increase in yield was observed when the plants were grown under eCO_2_ compared to aCO_2_ under HNT conditions. Among the genotypes, only DS25-1 showed a change in 100 seed weight under drought, causing a 15% reduction compared to the control (Fig. 6C), while DS31-243 was not influenced by either HNT or drought (Fig. 6D).

### Seed protein, oil, and carbohydrates

**Fig. 7:**
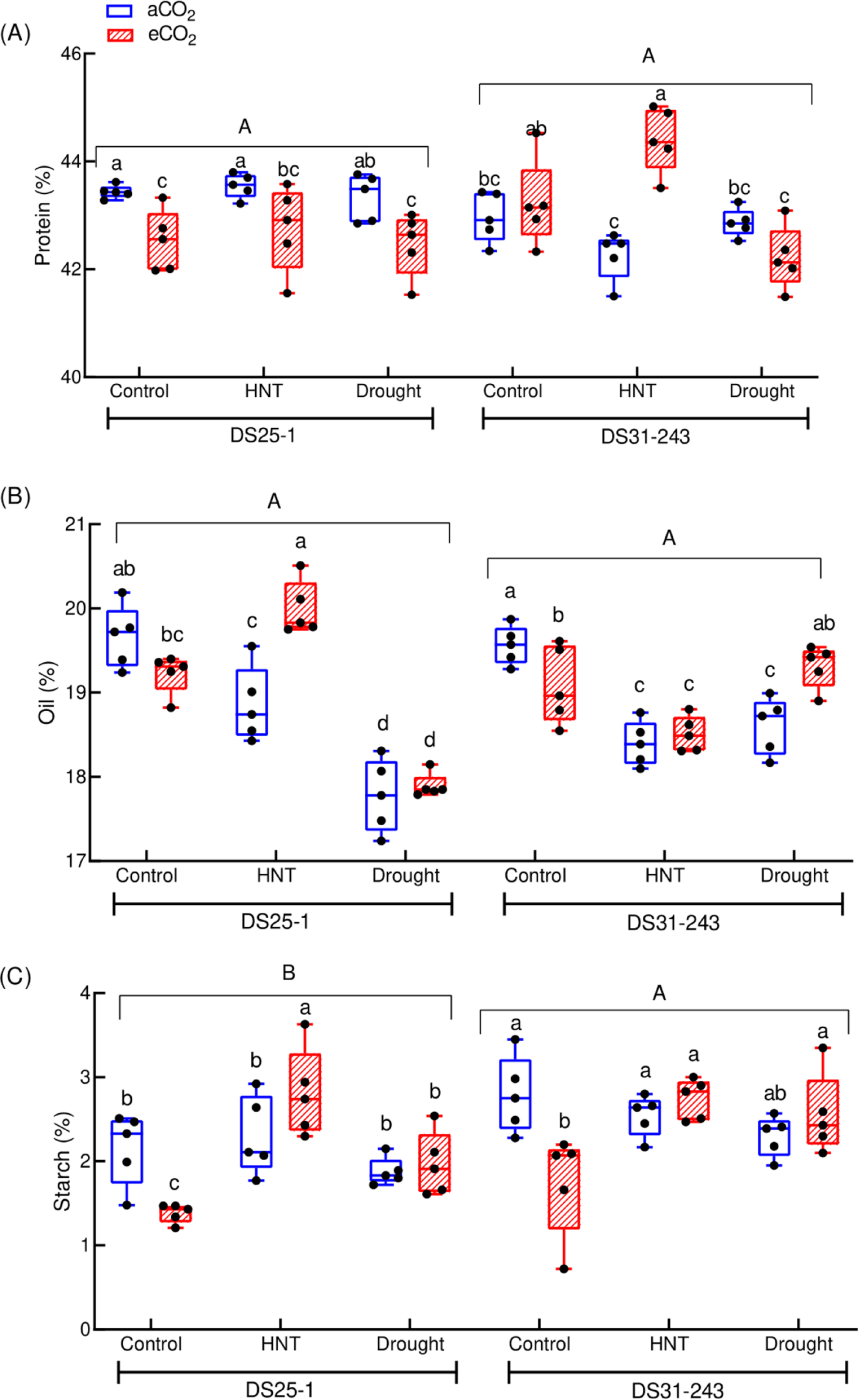
Effect of high-night temperature (HNT) and drought at ambient (aCO_2_) and elevated (eCO_2_) CO_2_ concentrations on (**A**) protein, (**B**) oil, and (**C**) starch content of DS25-1 and DS31-243 soybean genotypes. The upper and lowercase letters above the boxes are the least significant difference test (LSD) between genotypes and six treatments for each genotype. The treatment boxes with similar letters are statistically non-significant at *p* < 0.05.

A notable difference in seed protein, oil, and carbohydrate content was observed due to the treatments (*p* < 0.001; Table 2), while the genotypes were not significantly different for protein and oil. However, based on the partial ƞ^2^, the genotype had a 0.076 effect size on oil content (Table 5). Significant treatment × genotype interaction was observed for protein, oil, and carbohydrates, except starch and sucrose, while the effect size was large on all the quality traits. The treatments had a larger effect on all the measured quality traits. On average, the protein content of the seeds developed under eCO_2_ decreased by 2% under three growing conditions (control, drought, and HNT) in DS25-1 compared to control and aCO_2_. While it increased by 5% under HNT and eCO_2_ conditions compared to aCO_2_ in DS31-243 (Fig. 7A). The drought during the seed developmental stage did not alter the seed protein content in both genotypes. At aCO_2_, the oil content of DS25-1 and DS31-243 decreased under HNT by 4% and 6%, respectively, and under drought conditions by 9.5% and 5%, respectively, compared to control (Fig. 7B). The eCO_2_ increased the oil content of DS25-1 under HNT (6%) and DS31-243 under drought (4%).

**Table 5:** Partial Eta-squared (ƞ^2^) effect size and confidence interval (CI) of treatments, genotypes, and their interaction on seed quality traits.

Traits %	Sources	Partial ƞ^2^	Lower CI	Upper CI
Protein	Treatment	0.470	0.057	0.572
	Genotype	0.043	0.000	0.046
	Treatment × Genotype	0.538	0.206	0.627
Oil	Treatment	0.781	0.588	0.790
	Genotype	0.076	0.000	0.078
	Treatment × Genotype	0.736	0.472	0.817
Starch	Treatment	0.602	0.335	0.638
	Genotype	0.246	0.027	0.414
	Treatment × Genotype	0.180	0.006	0.194
Stachyose	Treatment	0.551	0.194	0.611
	Genotype	0.176	0.003	0.364
	Treatment × Genotype	0.282	0.021	0.385
Sucrose	Treatment	0.630	0.327	0.665
	Genotype	0.295	0.032	0.455
	Treatment × Genotype	0.213	0.030	0.245
Fructose	Treatment	0.585	0.093	0.694
	Genotype	0.818	0.584	0.853
	Treatment × Genotype	0.398	0.085	0.492
Glucose	Treatment	0.503	0.121	0.530
	Genotype	0.668	0.333	0.762
	Treatment × Genotype	0.302	0.059	0.408
Palmitic acid	Treatment	0.808	0.567	0.838
	Genotype	0.069	0.000	0.216
	Treatment × Genotype	0.528	0.181	0.636
Stearic acid	Treatment	0.649	0.185	0.752
	Genotype	0.533	0.194	0.666
	Treatment × Genotype	0.518	0.223	0.604
Linoleic acid	Treatment	0.586	0.241	0.663
	Genotype	0.037	0.000	0.106
	Treatment × Genotype	0.292	0.045	0.355
Linolenic acid	Treatment	0.347	0.066	0.375
	Genotype	0.042	0.000	0.152
	Treatment × Genotype	0.271	0.026	0.414
Oleic acid	Treatment	0.709	0.311	0.762
	Genotype	0.044	0.000	0.045
	Treatment × Genotype	0.474	0.123	0.577

**Fig. 8:**
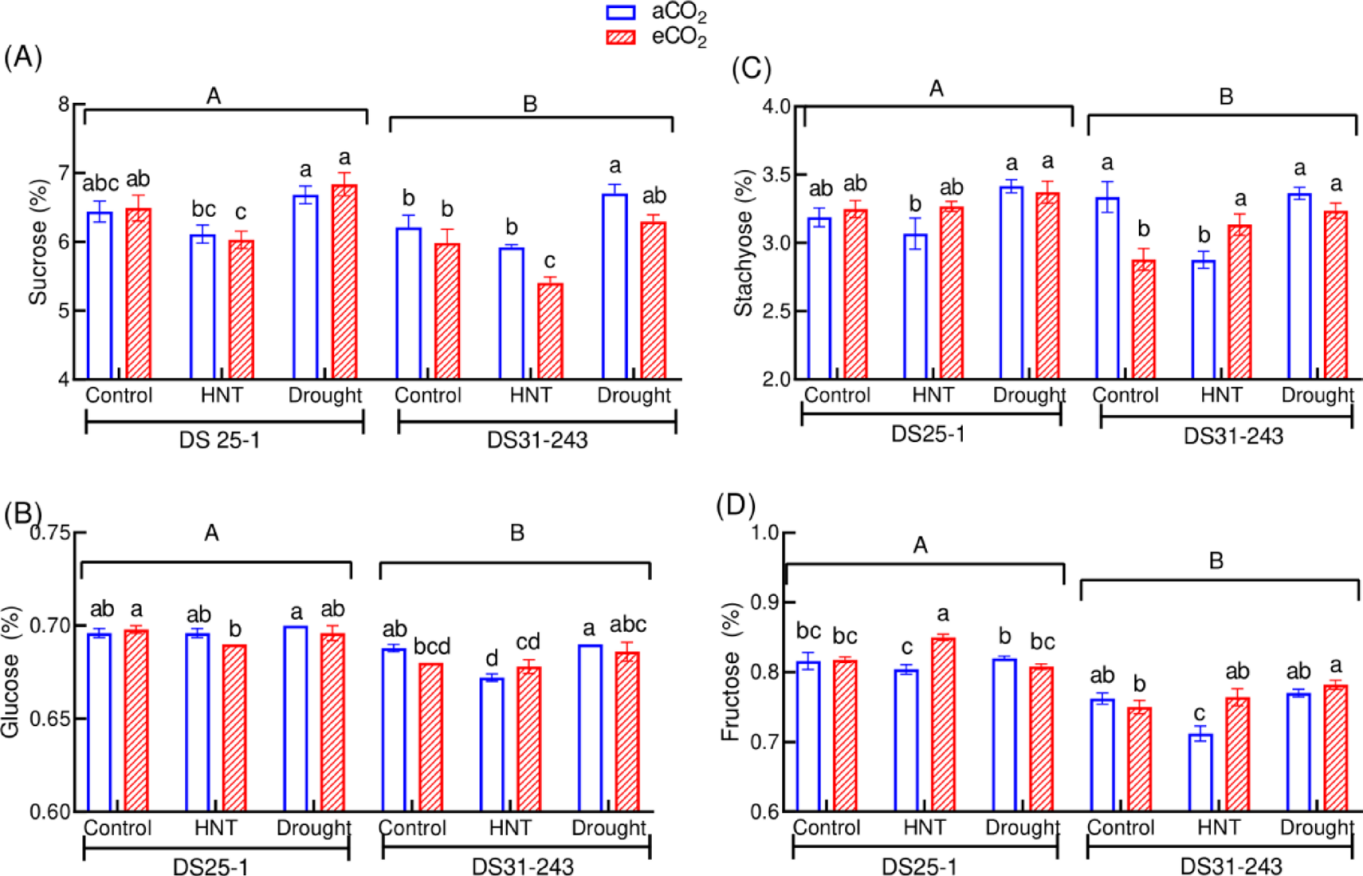
Effect of drought and high-night temperature at ambient (aCO_2_) and elevated (eCO_2_) CO_2_ concentrations on (**A**) sucrose, (**B**) glucose, (**C**) stachyose, and (**D**) fructose content of DS25-1 and DS31-243 soybean genotypes. The upper and lowercase letters above the boxes are the least significant difference test (LSD) between genotypes and six treatments for each genotype. The treatment boxes with similar letters are statistically non-significant at *p* < 0.05.

Under HNT conditions, the starch content of DS25-1 seeds increased by 22% at eCO_2_ compared to aCO_2_ (Fig. 7C). On the other hand, under control conditions, the starch content under eCO_2_ decreased by 3.6% and 3.7% in DS25-1 and DS31-243, respectively. The sucrose content of seeds was comparable between CO_2_ environments in all the three growing conditions in DS25-1, while the DS31-243 stored less sucrose at HNT at eCO_2_ (Fig. 8A). Even the glucose content of DS31-243 was lower at HNT (0.67%) compared to control (Fig. 8B). The drought condition during the seed development stage did not affect the stachyose content in both the genotypes providing on par result with the control (Fig. 8C). The stachyose content of DS31-243 seeds at HNT and aCO_2_ is significantly lower by 14% than control (aCO_2_), while it was on par under eCO_2_. Maximum fructose content was recorded under HNT and eCO_2_ conditions in DS25-1 (Fig. 8D). Under HNT conditions, the fructose content under eCO_2_ increased by 6 and 7% in DS25-1 and DS31-243, respectively, compared to aCO_2_.

### Fatty acid composition

**Fig. 9:**
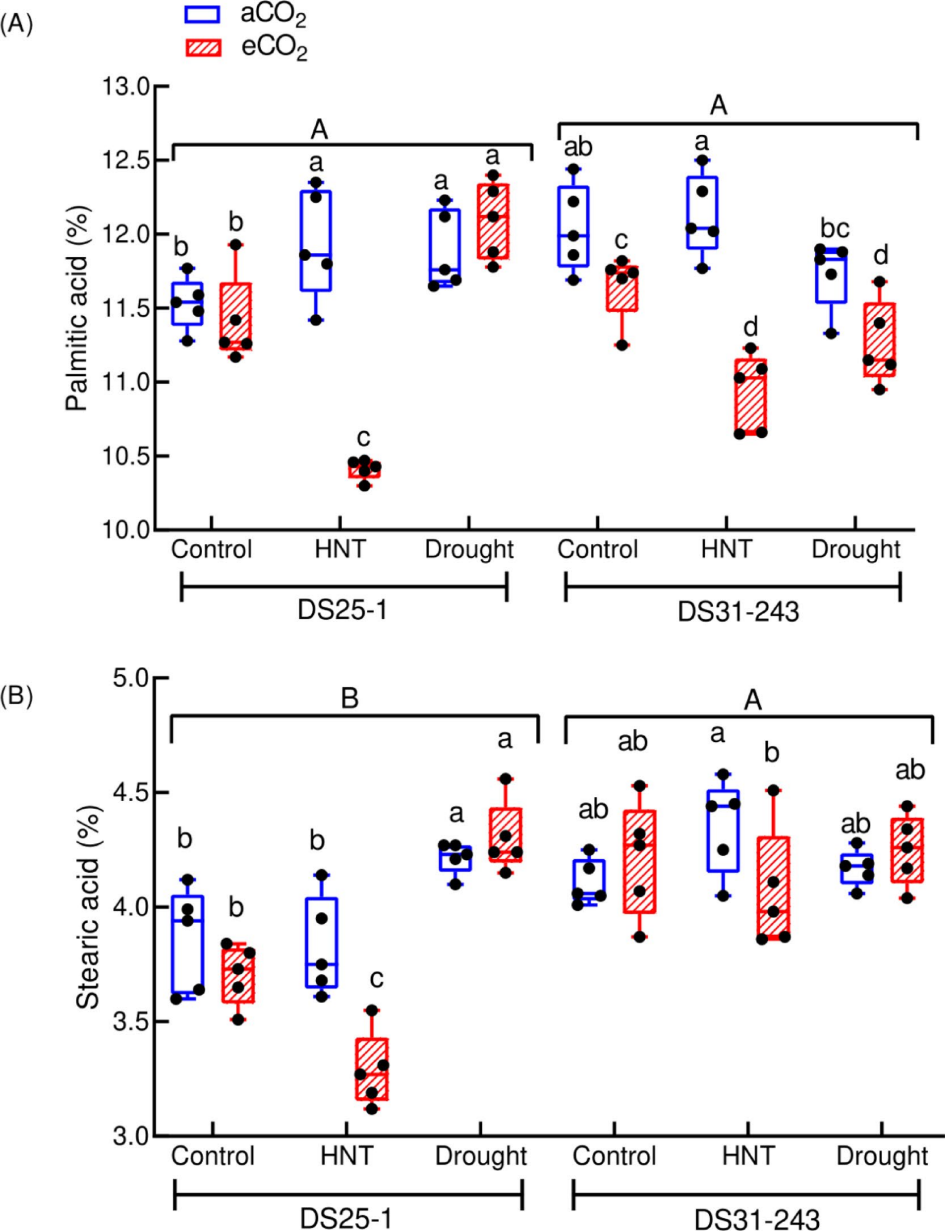
Effect of drought and high-night temperature at ambient (aCO_2_) and elevated (eCO_2_) CO_2_ concentrations on saturated fatty acid contents (**A**: Palmitic acid; **B**: Stearic acid) of DS25-1 and DS31-243 soybean genotypes. The upper and lowercase letters above the boxes are the least significant difference test (LSD) between genotypes and six treatments for each genotype. The treatment boxes with similar letters are statistically non-significant at *p* < 0.05.

The saturated and unsaturated fatty acids significantly differed for treatments (*p* < 0.05 and 0.001) (Table 2). Only stearic acid differed among the genotypes (*p* < 0.001). The effect size of treatments was larger than that of genotype and treatment × genotype interaction (Table 5). The genotypes had a smaller effect size on the unsaturated fatty acids. In DS25-1, the plants exposed to HNT under eCO_2_ had the lowest saturated fatty acids, both palmitic (10.41%) and stearic (3.3%) (Fig. 9A, B). However, in DS25-1, both saturated fatty acids increased under drought and eCO_2_ by 5% (palmitic) and 11% (stearic) compared to the control. Meanwhile, in the DS31-243, the palmitic acid content at HNT and drought under eCO_2_ decreased by 9% and 6%, respectively, compared to corresponding aCO_2_ treatments. The stearic acid content of DS31-243 decreased at HNT and eCO_2_ compared to HNT and aCO_2_ and was comparable across the remaining treatments.

**Fig. 10:**
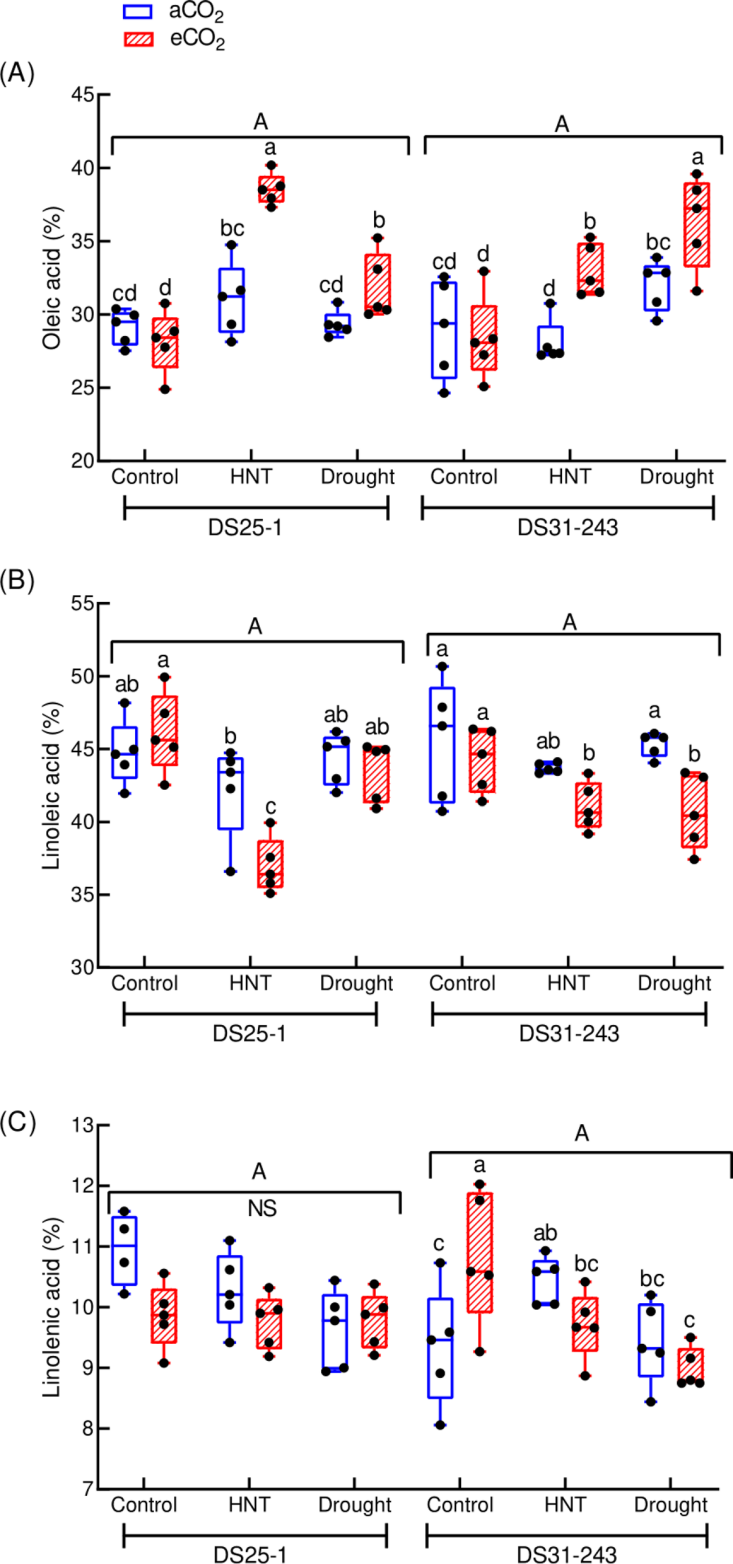
Effect of high-night temperature (HNT) and drought at ambient (aCO_2_) and elevated (eCO_2_) CO_2_ concentrations on unsaturated fatty acid contents (**A**: Oleic acid; **B**: Linoleic acid; **C**: Linolenic acid) of DS25-1 and DS31-243 soybean genotypes. The upper and lowercase letters above the boxes are the least significant difference test (LSD) between genotypes and six treatments for each genotype. The treatment boxes with similar letters are statistically non-significant. ‘NS’ indicates non-significant at *p* < 0.05.

The higher oleic acid content in DS25-1 was recorded under eCO_2_ conditions of HNT (32% higher than control at aCO_2_). In comparison, it was under eCO_2_ and drought conditions in DS31-243 (25% higher than control at aCO_2_) (Fig. 10A). During HNT, the eCO_2_ increased the oleic acid content by 24% and 17% in DS25-1 and DS31-243, respectively, compared to aCO_2_. Under drought conditions, the eCO_2_ increased the oleic acid content in both genotypes. The lowest linoleic acid content in DS25-1 was observed in HNT under eCO_2_ (37%) (Fig. 10B). The seeds of both genotypes developed under drought conditions at aCO_2_ did not change the linoleic acid content compared to the control. In the case of linolenic acids, the DS25-1 did not exhibit any significant difference between the treatments, neither under aCO_2_ nor under eCO_2_ (Fig. 10C). In DS31-243, eCO_2_ increased linolenic acid (16%) under control temperature and VWC conditions, compared to aCO_2_. At the same time, it did not induce significant changes under HNT and drought conditions. Additionally, under HNT, the linolenic acid increased by 12% compared to control under aCO_2_, whereas drought had no significant effect.

## Discussion

The intensity and shifts in the diurnal temperature range and projected drought risk conditions are becoming critical in beans and other crops^39^. The study delineated the effects of HNT and drought stress on the physiology, yield, and seed quality of soybean during flowering and seed developmental stages under current and elevated CO_2_ environments.

### eCO_2_ enhances photosynthesis under drought

Studies in soybeans have shown that reproductive and grain-filling stages are susceptible to drought or heat stress and their combinations^9,17^. Plants continuously respond to environmental changes to maintain homeostasis in carbon fixation and respiration^40^. It includes excess accumulation of reactive oxygen species (ROS) and upregulation of the antioxidant system, disrupted membrane permeability, changes in leaf gas exchange rates and subsequent reduction in photosynthetic rate, and increased photorespiration, among others^41–43^. These alterations in the plant leaves phenological changes with respect to biomass accumulation, partitioning, and yield^10^. As reported earlier, plants grown under eCO_2_ increased the photosynthetic activity of soybeans under control and drought conditions^44^. Drought-mediated reduction in RuBP carboxylase activity and subsequent reduction in photosynthetic efficiency^45^ is mitigated by eCO_2_ through enhanced gsw and internal CO_2_ concentration compared to plants grown under aCO_2_^46^. Though not monitored in the study, eCO_2_ improves the carbon sequestration rate via enhanced RuBP carboxylase activity, reducing the effects of drought on photosynthesis^47^. In addition, eCO_2_ increases the leaf mesophyll thickness, which reduces leaf transpiration and, thus, water loss in soybeans^47^. However, plants grown under HNT are not influenced by eCO_2_. The physiology of plants is comparable with control except for NPQ (Fig. 3). Contrary to the findings of Parkash et al.^48^, which reported no change in NPQ under high night temperature conditions in cotton, our study observed an increase in NPQ under 30/26 °C. Under eCO_2_ conditions, the NPQ increased under all three treatment conditions of control, HNT, and drought. While the statistical analysis revealed no significant differences between the genotypes for the measured physiological traits, it exhibited a small to medium effect size due to genotype. Unlike previous reports on the reduction of photosynthesis and other physiological parameters under HNT, our results did not find any significant differences compared to the control, which may be due to the narrow diurnal temperature range followed by other researchers^22,49^. It has been reported that the rate of nighttime warming is 20% higher than the daytime rate per century^4,50^. During summer, the average nighttime temperature of Mississippi and Texas is 22 °C and 23 °C, with a range of 18–24 °C. Thus, HNT treatments with 29 and 30 °C temperatures are far from accurate. In addition, the growing conditions, such as growth chambers and greenhouses, restrict the availability of light required for carbon assimilation, making it challenging to estimate the effect of HNT.

### Seed yield and biomass were reduced under drought, while they increased at HNT and eCO_2_ conditions

Plants exposed to an HNT temperature of 30/26 °C during flowering and seed developmental stages increased the plant biomass, pods, and seeds compared to the control temperature of 30/22 °C. Previous studies of HNT on various crops reported that the plant either exhibits no response^51^ or responds with a reduction in plant growth and seed yield^31,49,52^. However, in our study, the genotype DS25-1 under HNT exhibited a comparable response to the control at aCO_2_, while DS31-243 reduced the seed yield by 42% compared to the control temperature at aCO_2_. On the other hand, under eCO_2_, the genotypes performed better under HNT. Hence, the DS25-1 genotype is better suited for cultivation under HNT conditions and can be used in the breeding program as a tolerant source. The vegetative and reproductive development of crops increases with an increase in temperature up to the species’ optimum level^53^. The average optimum temperature of soybean for vegetative and reproductive growth is within the range of 27–30 °C^54^. In addition, the indeterminate genotypes of soybean add more nodes when grown under high temperatures^55^. These results align with the findings of our study, which manifest increased plant height and node number under HNT in both genotypes. The subsequent increase in stem weight and number of flowering sites increased the total plant biomass as well as the number of pods and seeds. It has been reported that the increased early daytime stomatal conductance because of HNT helped to gain biomass and grain yield in wheat^56^. The study observed a 43% increase in yield with a 19% rise in early daytime stomatal conductance under HNT over control. In soybean, the high day and nighttime temperatures of 38/30 and 42/34 °C incorporated 32 and 38 nodes compared to 23 under 30/22 °C optimum temperature^55^. Gibson and Mullen^51^ observed that an HNT of 30 °C increased the number of pods and seeds, reduced the seed size, and did not affect the seed yield of soybean compared to the control temperature of 20 °C. Zheng et al.^57^ observed that the HNT stimulates flower opening and pod setting on secondary and tertiary racemes, increasing the number of pods per plant. In this study, the number of pods and seeds of DS25-1 increased under HNT, while the DS31-243 had lower but comparable counts with the control under both aCO_2_ and eCO_2_. This lower count of seeds and pods might have contributed to the reduced seed yield in DS31-243 under HNT at aCO_2_. It highlights the reproductive success of DS25-1, showing resilience under HNT conditions. It is important to note that HNT did not influence the 100 seed weight of both DS25-1 and DS31-243 under both CO_2_ environments. Additionally, the average daily temperature in our HNT treatment (30/26 °C) was 28 °C, falling within the optimum temperature range for soybeans. In addition, the plants were provided with optimum water and nutrients in a sunlit plant growth chamber, which offsets other growth limitations affecting the plant. The carbon fertilization effect of eCO_2_ complemented the response of plants to HNT, resulting in increased seed yield compared to aCO_2_ and control.

On the contrary, the plants grown under drought conditions decreased the yield by 62% and 56% in DS25-1 and DS31-243, respectively. It has been reported that the key metabolic functions of plants exposed to drought conditions during critical growth stages, like flowering and seed development, are negatively impacted, leading to lower plant performance in terms of biomass accumulation and seed yield^58^. Drought-induced production of ROS impairs the plant metabolic processes^59^, causing cell membrane damage, damage to protein and nucleic acids, inhibition of stomata closure, and associated photosynthetic and enzyme activities^60,61^. In our study, seed yield parameters such as the number of pods and seeds, and 100-seed weight decreased under drought. Similar observations were made in the past studies^13,61^. Though a reduction of 42 and 47% of total dry weight was observed in DS25-1 and DS31-243, respectively, the biomass allocation to roots was not limited under drought conditions (Fig. 5). In fact, the root dry weight of plants grown under drought was on par with the plants under control at similar CO_2_ conditions in both cultivars. Root systems are crucial for developing drought-tolerant crops, as they are the first plant organs to detect signs of drought and changes in water potential^62^. Breeding efforts often focus on root architectural traits to create crop varieties that can better adapt to water-limited conditions^63^. Roots adjust their structure to optimize resource uptake while minimizing metabolic costs. Under prolonged drought, roots extend deeper into the soil to access water and nutrients^64^. Among the two genotypes, DS31-243 exhibited relatively higher root biomass under drought conditions compared to DS25-1. Allowing the plants to absorb water and nutrients from deeper soil layers to tolerate the drought severity. This might have contributed to a relatively lower reduction in seed yield in DS31-243 than in DS25-1. Hence, the DS31-243 can be used in the breeding program as a source for drought tolerance. It is worth noting that the 100-seed weight of DS25-1 significantly reduced under drought, while it remained unchanged in DS31-243, revealing its potential in breeding. The carbon fertilization of plants under drought during flowering and seed developmental stages did not produce better biomass or seed yield. Though we observed enhanced photosynthetic performance due to eCO_2_ under drought, it did not translate into seed yield, unlike previous studies^47,65^.

### Seed oil content decreased while oleic acid increased under stress conditions

Seeds developed under HNT and drought conditions exhibited altered seed quality composition, and eCO_2_ modified their effects. The seed protein content of the DS25-1 genotype was notably reduced by eCO_2_ under control and drought conditions. This observation in the reduction of protein can be attributed to the dilution effect of eCO_2_^66^. It has been proposed that crops with C_3_ photosynthesis, such as wheat, rice, and peas, experience a reduction in seed protein content under eCO_2_ conditions^67^. Depending on the cultivars, as much as a 2 to 6% reduction in seed protein content was previously reported in soybeans under eCO_2_^66^. However, the genotype DS31-243 produced more seed protein under HNT conditions, exhibiting genetic variability of plant response to eCO_2_ and its usefulness as a genetic source for quality seed production under HNT conditions. Compared to DS25-1, the average oil content in DS31-243 was lower under HNT than under control conditions at eCO_2_. This suggests that the protein-oil trade-off in the seed contributed to the increase in protein content under HNT and eCO_2_ in DS31-243. Contrary to protein, the oil content of genotypes decreased when grown under HNT and drought conditions compared to the control. The HNT and drought-induced reduction in seed oil content are reported in soybean^17,31,68^, which is attributed to the limited availability of substrates. Carbohydrates were mostly affected by HNT than by drought. The carbohydrate content of seeds exposed to drought conditions was on par with the control in both genotypes. Under HNT conditions, the plant’s exposure to eCO_2_ increased the starch content in DS25-1. Increased photosynthate supply under eCO_2_ conditions, coupled with favorable temperature, has resulted in increased starch accumulation in this genotype. On the other hand, the observed reduction in sucrose, stachyose, glucose, and fructose content under HNT is associated with increased nighttime respiration^31,69^. In addition, the reduced duration of the stay-green window during the HNT condition due to early leaf senescence decreases the carbohydrate levels required for seed filling^69^.

The combination of eCO_2_ with HNT and drought influences the saturated and unsaturated fatty acids of seeds. In both genotypes, the palmitic and stearic acid content decreased under HNT and eCO_2_ conditions, while the monounsaturated fatty acid and oleic acid content increased. This could be due to the alteration in the carbon and nitrogen ratio and alteration in the activity of the desaturase enzyme due to eCO_2_ conditions^70,71^. In accordance with previous results, the linolenic and linoleic acid content was lower under stressful conditions compared to oleic acid^72^. The reduced polyunsaturation was attributed to the activation of the triacylglycerol degradation pathway, especially the decreased activity of the desaturase enzyme under stress^70^. This noted a reduction in polyunsaturated fatty acids and subsequent reduction in unsaturation under high temperature and drought conditions during early seed developmental stages is a stress-tolerant mechanism to achieve membrane stability and protect the cells from fatty acid oxidation^73^.

## Conclusion

The study provides insight into the effect of drought and HNT under ambient and elevated CO_2_ environments. Overall, the physiology of the plants was affected when grown under HNT and drought conditions during the reproductive and seed-filling stages. The plants grown under drought conditions during the reproductive and seed-filling stages showed lower plant biomass in both genotypes. Subsequently, the drought conditions during the reproductive stage resulted in a significant reduction in seed yield. The plants grown under HNT of 30/26 °C during reproductive and seed development stages showed lower seed yield in DS31-243, while it did not affect DS25-1. This study suggests that DS25-1 is better suited for HNT conditions under aCO_2_. On the contrary, the eCO_2_ during the HNT conditions positively impacted the biomass accumulation and seed yield of both genotypes. The study observed a reduction in protein content under eCO_2_, a reduction in carbohydrate content under HNT, and decreased polyunsaturation in the seeds under both HNT and drought conditions. The positive impact of HNT observed in the study was due to the low-temperature range, which falls within the daily average optimum temperature range of the soybean current production system. Since the study was carried out in sunlit plant growth chambers under optimum nutrient supply, the results may not fully reflect plant responses under natural growing conditions. Therefore, these findings should be further validated under field conditions to gain a more comprehensive understanding of plant behavior under ambient conditions. This step will help ensure that the results obtained in controlled environments are applicable to real-world scenarios. Field trials can uncover factors that may not be evident in a controlled setting, such as soil type, pest pressures, and weather variations, thereby helping to optimize crop management practices. Furthermore, screening multiple genotypes that represent a range of growth habits and maturity groups is essential to identify genetic sources of drought tolerance and high nighttime temperature tolerance. This approach can significantly contribute to soybean crop improvement programs by integrating resilient traits into breeding efforts.

## Data Availability

Data is included in the manuscript.
